# A review of TSHR- and IGF-1R-related pathogenesis and treatment of Graves’ orbitopathy

**DOI:** 10.3389/fimmu.2023.1062045

**Published:** 2023-01-19

**Authors:** Xuejiao Cui, Futao Wang, Cong Liu

**Affiliations:** ^1^ Department of Endocrinology, Shengjing Hospital of China Medical University, Shenyang, China; ^2^ Department of Endocrinology, Changchun Central Hospital, Changchun, China

**Keywords:** Graves’ orbitopathy, orbital fibroblasts, orbital adipocytes, thyrotropin receptor, insulin-like growth factor-1 receptor

## Abstract

Graves’ orbitopathy (GO) is an organ-specific autoimmune disease, but its pathogenesis remains unclear. There are few review articles on GO research from the perspective of target cells and target antigens. A systematic search of PubMed was performed, focusing mainly on studies published after 2015 that involve the role of target cells, orbital fibroblasts (OFs) and orbital adipocytes (OAs), target antigens, thyrotropin receptor (TSHR) and insulin-like growth factor-1 receptor (IGF-1R), and their corresponding antibodies, TSHR antibodies (TRAbs) and IGF-1R antibodies (IGF-1R Abs), in GO pathogenesis and the potentially effective therapies that target TSHR and IGF-1R. Based on the results, OFs may be derived from bone marrow-derived CD34+ fibrocytes. In addition to CD34+ OFs, CD34- OFs are important in the pathogenesis of GO and may be involved in hyaluronan formation. CD34- OFs expressing Slit2 suppress the phenotype of CD34+ OFs. β-arrestin 1 can be involved in TSHR/IGF-1R crosstalk as a scaffold. Research on TRAbs has gradually shifted to TSAbs, TBAbs and the titre of TRAbs. However, the existence and role of IGF-1R Abs are still unknown and deserve further study. Basic and clinical trials of TSHR-inhibiting therapies are increasing, and TSHR is an expected therapeutic target. Teprotumumab has become the latest second-line treatment for GO. This review aims to effectively describe the pathogenesis of GO from the perspective of target cells and target antigens and provide ideas for its fundamental treatment.

## Introduction

Graves’ orbitopathy (GO), also known as thyroid eye disease and thyroid-associated ophthalmopathy, is relatively rare, with an incidence of 0.54-0.9 cases/100,000/year in males and 2.67-3.3 cases/100,000/year in females (1). Approximately 25%-50% of patients with Graves’ disease (GD) have GO, with the incidence varying based on the testing tool used to diagnose GO, such as clinical signs and symptoms, physical examination, and imaging studies. Common symptoms of GO include eye pain, photophobia, blurred vision, excessive tear production and double vision. GO can cause periorbital oedema, exophthalmos, eyelid recession and changes in eye movement, affecting one or both eyes. Approximately 5–6% of patients with GO have severe disease with compressive optic neuropathy or sight-threatening corneal ulceration, and these patients may experience vision loss. GO occurs at any age, but women 30-50 years of age are most commonly affected (2). Nevertheless, the severity of GO tends to be worse in men and in patients who are first diagnosed when they are older than 50 years old (3). Traditional risk factors for GO include smoking, hypercholesterolaemia, thyroid dysfunction, radioiodine therapy, high thyrotropin receptor antibodies (TRAbs), and low thyroid peroxidase antibodies (TPOAbs) and thyroglobulin antibodies (TgAbs). New risk factors for GO include high thyroglobulin (Tg) (4), selenium deficiency, intestinal flora imbalance, and increased levels of both *Yersinia enterocolitica* and *Escherichia coli* in the digestive tract (5).

GO is characterized by inflammation in retrobulbar tissues, increased adipogenesis, and accumulation of extraocular intramuscular glycosaminoglycans (GAGs), which result in expansion and remodelling of the orbital contents (6, 7). The pathogenesis of GO is unclear. Orbital fibroblasts (OFs) and orbital adipocytes (OAs) are important cells that are targeted by the autoimmune response, and thyrotropin receptor (TSHR) and insulin-like growth factor-1 receptor (IGF-1R) are key target antigens. Moreover, increased production of TRAbs is associated with the prevalence and severity of GO. This review describes recent advances in GO research related to primary target cells (OFs and OAs), primary target antigens (TSHR and IGF-1R) and associated antibodies, and targeted antigen-specific therapies for GO. Data acquisition was based on PubMed search strategies, with a particular focus on papers published after 2015.

## Immune cells and GO

Both cellular and humoral immunity play important roles in the pathogenesis of GO. High expression of HLA-DR and adhesion molecules on the vascular endothelium of the orbital tissues of GO patients leads to strong infiltration of orbital immune cells, such as dendritic cells (DCs), macrophages, mast cells, B lymphocytes and T lymphocytes. Most of the current evidence indicates that the level of T and B lymphocyte infiltration correlates with GO disease activity (8, 9). Antigen-presenting cells (APCs), such as DCs and macrophages, present TSHR to CD4+ T lymphocytes in the context of MHC-II molecules. Subsequently, CD4+ T lymphocytes release cytokines to activate CD8+ T lymphocytes or autoantibody-producing B lymphocytes, and a cascade of effects amplifies the immune response and promotes the autoimmune process. CD4+ T lymphocytes are the main cells dominating orbital inflammatory infiltration and are classified into various subtypes, including T helper (Th) 1, Th2, Th17, and Treg cells (10). Different subgroups of T lymphocytes play dominant roles in different stages of GO. Th1 cells, which produce interleukin (IL)-1β, IL-2, TNF-α, and IFN-γ, induce a cell-mediated immune response in the early stage, whereas Th2 cells, which release IL-4, IL-5, IL-10, and IL-13, activate humoral reactions and promote the production of IgG in the late stage. A recent study showed that increased concentrations of Th2 chemokines (CCL2) in plasma from patients with GO may reflect disease activity (11). Additionally, Fang et al. (12) reported that compared with healthy control individuals, patients with GO have significantly higher levels of IL-17A-producing T lymphocytes and recruitment of both CD4+ and CD8+ T lymphocytes in the orbits. Furthermore, orbital tissues from patients with GO express more IL-17A receptor, IL-17A and its related cytokines, with severe fibrotic changes, compared with healthy control individuals. Another study by Fang et al. (13) showed that IFN-γ- and IL-22-expressing Th17 cells are increased in patients with GO, which was positively related to the clinical activity score (CAS), and that IL-17A promotes TGF-β-induced fibrosis in CD90+ OFs from patients with GO (GO-OFs). These findings suggest the potential pathogenic role of Th17 cells in the inflammatory response and fibrosis associated with GO. However, uncertainty remains as to whether Treg cell levels correlate with the severity and activity, clinical course, or treatment response of GO (14, 15).

## Target cells of GO

### Orbital fibroblasts

OFs are target cells of the autoimmune response in GO. They participate in proliferation, adipogenesis and overproduction of extracellular matrix GAGs, including hyaluronan (HA), which are involved in GO pathogenesis (16). Recently, reported proteomics and DNA methylation data indicate that OFs from patients with active GO are involved in inflammation, adipogenesis, and GAG production, and OFs from patients with inactive GO are more inclined to play an active role in extracellular matrix remodelling (17). MicroRNAs have been reported to be involved in HA production. The results of one study indicated that overexpression of miR-146a reduces the production of HA and collagen I in GO-OFs, and it was demonstrated that miR-146a downregulates the secretion of HA and collagen I in GO-OFs *in vitro* (18). A recent study showed that the LPAL2/miR-1287-5p axis modulates TGF-β1-induced increases in cell adhesion factor levels and GO-OF activation *via* EGFR/AKT signalling (19). OFs express MHC-II molecules (20, 21), and the study showed that GO-OFs present their own antigens to T lymphocytes *via* MHC-II molecules and employ CD40-CD40 L signalling, leading to activation of T lymphocytes and further stimulating fibroblast proliferation. According to the literature, most OFs are positive for CD90 (Thy-1) expression and negative for CD45 expression (22); these proteins are positive and negative markers of mesenchymal stem cells, respectively. Activated OFs differentiate according to the expression of Thy-1 on their cell surface. Thy-1+ OFs are mainly present in extraocular muscles; they differentiate into myofibroblasts and overproduce HA. This leads to extraocular muscle oedema and enlargement. A recent study showed that simvastatin inhibits TGF-β-induced myofibroblast differentiation by inhibiting the RhoA/ROCK/ERK and p38 MAPK signalling pathways, suggesting that simvastatin is a potential therapeutic drug for the prevention and treatment of GO orbital fibrosis (23). Thy-1- OFs, which are called preadipocytes, are mainly present in connective tissue and differentiate into adipocytes (24); Thy-1+ OFs suppress adipocytic differentiation of Thy-1- OFs by producing antiadipocytic factors. Therefore, the balance and relative proportion of Thy-1- and Thy-1+ OFs modulate tissue remodelling in GO (25).

Bone marrow-derived CD34+ fibrocytes are a monocyte subpopulation of peripheral blood mononuclear cells that infiltrate orbital tissues and promote the onset of GO (26). A recent study showed that the concentration of circulating fibrocytes is significantly higher in patients with GO than in patients with GD and healthy control individuals, and in GO patients, these fibrocytes express a significantly higher level of TSHR (27). CD34+ OFs have been reported to express major thyroid autoantigens, including TSHR, thyroperoxidase (TPO), thyroglobulin (Tg) and sodium-iodide symporter (NIS) (28). In response to inflammatory factors, CD34+ OFs further differentiate into myofibroblasts or adipocytes (29).

CD34- OFs coexist with residential CD34+ OFs, but little was previously known about the role of CD34- OFs until CD34- OFs and CD34+ OFs were recently sorted separately by cytometry. The expression of cytokines and HA synthases (HASs) differs in these cell subsets. HAS1, HAS2, and HAS3 are differentially inducible in various cell types by several cytokines and growth factors (30). These enzymes differ from each other in catalytic activities (HAS3 > HAS2 > HAS1) and the sizes of their final products. HAS1 and HAS2 polymerize long stretches of GlcAGlcNAc disaccharide chains, whereas HAS3 polymerizes relatively short stretches (<300 kDa) (31). IL-12p35 is mainly expressed in CD34- OFs, and IL-23p19, IL-6 and TNF-α levels are higher in CD34+ OFs (32, 33). Basal and TSH-induced HAS1 expression occurs in CD34+ OFs, and HAS2 and UDP-glucose dehydrogenase are mainly expressed by CD34- OFs (33). HAS2 appears to be responsible for HA synthesis in both cell subpopulations, and the relatively low levels of HAS2 in CD34+ OFs appear to represent the basis for the substantially higher levels of HA synthesized in CD34− OFs (33). Slit2, an axon guidance glycoprotein, is expressed and released by CD34- OFs and dampens the inflammatory phenotype of CD34+ OFs. Specifically, rhSlit2 dramatically represses the expression of TPO, Tg, TSHR, AIRE (Autoimmune regulator) and NIS and represses that of TNF-α, IL-6, and IL-23 induced by TSH in CD34+ OFs (33, 34). Slit2 knockdown enhances HAS1 expression in CD34- OFs but reduces basal and TSH-dependent HAS2 expression (33). Hence, Slit2 may represent a factor that was previously unrecognized for its capacity to modulate immune responses and HA synthesis in human tissues.

### Orbital adipocytes

**Figure 1:**
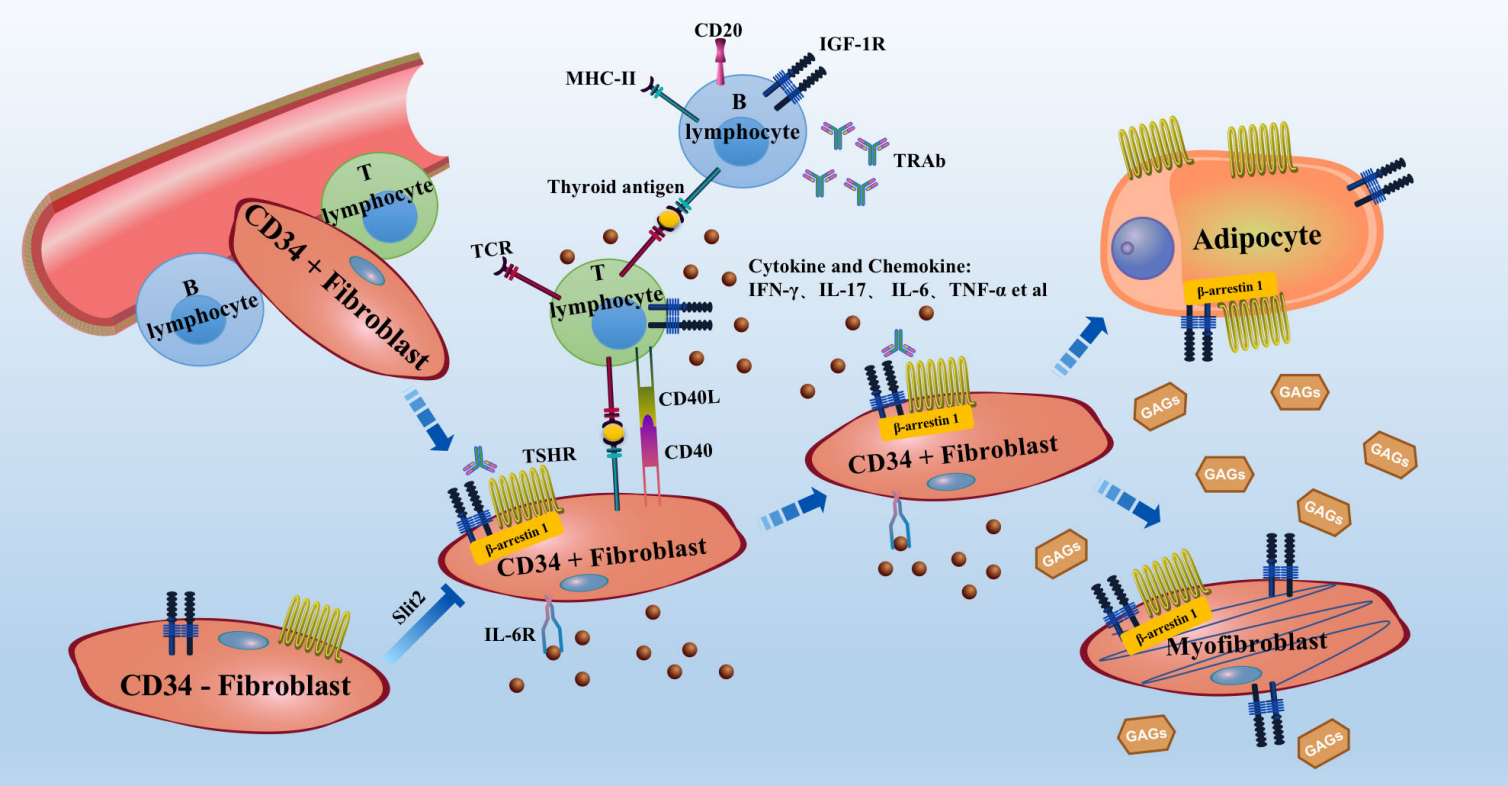
Schematic of the theoretical pathogenesis of GO Bone marrow mononuclear cell-derived CD34+ fibroblasts enter the orbit from the circulation and express low levels of TSHR, thyroglobulin and other thyroid antigens. APCs, such as B lymphocytes or macrophages, present antigens to antigen-specific T lymphocytes and activate them. Activated T lymphocytes release cytokines and chemokines, further causing B lymphocytes to produce antibodies. These processes form an inflammatory microenvironment in the orbit. OFs can also act as APCs. In the inflammatory microenvironment, activated T lymphocytes activate OFs. Activated OFs further secrete cytokines and chemokines and produce excessive levels of GAGs, leading to swelling of the orbital tissue. CD34+ fibroblasts are found in orbit with CD34− fibroblasts. CD34+ fibroblasts further differentiate into myofibroblasts or adipocytes, resulting in thickening of the extraocular muscles and exophthalmos. TSHR expression is increased during adipogenesis. IGF-1R, TSHR, IL-6R, CD20 and TNF-α are current therapeutic targets for GO. APC, antigen-presenting cell; GAG, glycosaminoglycan; GO, Graves’ orbitopathy; OFs, orbital fibroblasts; TSHR, thyrotropin receptor; IGF-1R, insulin-like growth factor-1 receptor.

Adipogenesis contributes to orbital adipose tissue expansion. A fibroblast has an approximate diameter of 30 microns; the diameter of a mature adipocyte is approximately 150 microns, which is 5 times larger. According to the literature, increased expression of TSHR during adipogenesis and binding of TRAb to TSHR on adipocytes leads to upregulation of HAS gene expression and excessive production of HA (35). Signalling pathways, including those for cAMP, PI3K-AKT, AGE-RAGE, lipolysis regulation, and thyroid hormone, are enriched in orbital fat isolated from patients with GO. The IGF-1R and Wnt signalling pathways appear to be enriched early in adipogenesis (36), and Wnt signalling inhibits adipogenesis in GO-OFs (37). PI3K can be activated by TSH, IGF-1, or multiple cytokine receptors, resulting in AKT activation. Activated AKT inhibits FOXO1 and activates mTOR to promote HA production and adipogenesis *via* peroxisome proliferator activator gamma (PPARγ) (38). PPARγ belongs to the nuclear receptor family of transcription factors and is expressed in adipocytes; it acts as a transcription factor and regulates the homeostasis of lipids and glucose. A recent study demonstrated that the mRNA level of protein kinase RNA-like endoplasmic reticulum kinase (PERK) is significantly higher in orbital tissues from patients with GO than in those from patients without GO. The expression of PPARγ is downregulated and oxidative stress and adipogenesis are reduced in PERK siRNA-transfected GO-OFs (39). One study showed that the expression of glycogen synthase kinase-3β (GSK-3β) in GO orbital tissues is significantly higher than that in control orbital tissues. During adipocyte differentiation, fibroblasts treated with the GSK-3β inhibitor CHIR 99021 showed decreased lipid droplets and decreased expression of PPARγ and c/EBPα and -β. Moreover, inhibition of Wnt and β-catenin in adipogenesis is reversed by CHIR 99021 (40). MicroRNAs have also been reported to be involved in adipogenesis; miR-130a is upregulated in Thy-1-OFs, inhibits AMPK activation, and promotes lipid accumulation in GO-OFs, leading to excessive fatty tissue accumulation in the orbit (41). Another recent study demonstrated that TGF-β-treated human placental mesenchymal stem cells (hPMSCs) suppress adipogenesis and lipogenesis in GO-OFs and in GO mice; the effects are mediated by the SMAD 2/3 pathways, suggesting that these cells may be a new and safe method to promote the antiadipogenic function of hPMSCs to treat GO (42). HA overproduction and adipogenesis have been observed in fibroblasts from orbital adipose tissue (OAT) but not in those from white adipose tissue (WAT) (43). This finding can be explained by the fact that human OAT is derived from the neural crest in the orbit (44) but that WAT is derived from the mesoderm (45). A schematic of the theoretical pathogenesis of GO is shown in **Figure 1**.

## Autoantigens in the pathogenesis of GO

### TSHR

TSHR is a G protein-coupled receptor (GPCR) that contains 3 domains (an ectodomain, transmembrane domain, and intracellular domain). It is composed of an A subunit, comprising a large extracellular domain, and a B subunit, which consists of a short extracellular fragment anchored in the cell membrane and an intracellular part. TSHR is a common target autoantigen of GD and GO (46). A new preclinical model of GO has been established in female BALB/c mice. This mouse model was established *via* immunization with the human TSHR α subunit by electrotransfection of a plasmid expressing human TSHR. This model exhibits infiltration of a large area around the optic nerve, increased adipogenesis, orbital muscle hypertrophy, extraocular muscle hypertrophy, and conjunctival oedema (47). TSHR is expressed in thyroid cells, which regulate thyroid function, and studies have shown that preadipocyte fibroblasts and myofibroblasts in the orbital tissues of patients with GO also express TSHR (48, 49). TSHR activation of OFs by signalling through cAMP–protein kinase A (PKA) to cAMP response element-binding protein-binding sites in the promoters of HAS1 and HAS2 increases HA production (50). mRNA expression of TSHR in orbital fat/connective tissue in patients with active GO is higher than that in patients with inactive GO, with the levels being directly related to mRNA levels of IL-1β (51). The expression level of TSHR in fibroblasts has been found to be much lower than that in thyroid cells. Indeed, one study showed that TSHR levels are 11-fold higher in GO or control thyrocytes than in fibroblasts (52). The exact mechanism by which TSHR, a specific antigen in thyroid cells, is ectopically expressed in the orbital tissues of patients with GO and becomes the target of the immune response is still unclear. However, this outcome likely occurs because OFs originate from monocyte progenitor cells in the bone marrow and have the ability to express TSHR (49). It has also been shown that preadipocyte fibroblasts express TSHR after stimulation with TSH, TRAb, and IL-6, and the adenylate cyclase/cAMP and PI3K/pAKT pathways become activated. Thus, the differentiation of fibroblasts to adipocytes and HA production are promoted (53). The findings of Woeller et al. (54) also demonstrate that TSHR signalling in GO-OFs stimulates proliferation indirectly through induction of miR-146a and miR-155, reducing the expression of ZNRF3 and PTEN that normally block cell proliferation. The latest interesting study by Draman MS et al. suggests a variant in TSHR that might explain the antigenic role of this receptor in OF even prior to adipogenesis (55). In conclusion, TSHR plays a specific role in GO pathogenesis and has become a therapeutic target.

### IGF-1R

Although data show that TSHR plays an important role in GO, IGF-1R may also have a key function. IGF-IR is a membrane-spanning receptor tyrosine kinase (RTK) that binds IGF-I and IGF-II and activates two important downstream signalling pathways, MAPK and PI3K/AKT. Evidence indicates that compared with a normal control, GO-OFs exhibit increased HA synthesis after IGF-1 treatment. Pretreatment with an IGF-1R blocking monoclonal antibody (1H7) or AKT inhibitor significantly decreased the HA concentration. IGF-1 treatment increases the level of pAKT expression; 1H7 and PI3K blockers decrease the expression of PI3K and pAKT protein, and AKT inhibitors decrease the expression of PI3K, AKT and pAKT (56). A study has shown that IGF-IR is overexpressed by OFs and T and B lymphocytes in patients with GO; IGF-1R regulates HA synthesis and adipogenesis in the orbit and defines the phenotype and function of T and B lymphocytes (57). A recent study indicated that 1H7 inhibits autophagy and induces apoptosis by suppressing IGF-1R signalling. This leads to retro-OF/OA death, which may be the main mechanism by which teprotumumab (a human monoclonal IGF-1R blocking antibody) reduces ocular protrusion in patients with GO (58). Teprotumumab has also been reported to attenuate constitutive expression and induction by TSH of MHC-II and B7 family members, including CD80, CD86, and programmed death-ligand 1 in CD34+ fibrocytes (59). At present, teprotumumab is a second-line treatment for GO, which is discussed in detail below.

### TSHR and IGF-1R complex

Although TSHR and IGF-1R are two independent receptors, there is a certain connection.

A study in an animal model showed that mice can develop a hyperthyroid state with inflammation and fibrosis of the orbital tissue after being transfected with plasmids expressing IGF-1Rα and TSHR (60). Tsui et al. (52) found that TSHR and IGF-1R colocalize by performing confocal microscopy of OFs and cultured human thyroid epithelial cells. Simultaneous activation of TSHR and IGF-1R causes rapid, synergistic phosphorylation/activation of ERK1 and ERK2 in primary cultures of GO-OFs and human thyrocytes as well as human embryonic kidney (HEK) 293 cells overexpressing TSHR (61). Tramontano et al. (62) reported a possible interaction between IGF-1 and TSH and that IGF-1 enhances the proliferation and DNA synthesis of FRTL-5 thyroid epithelial cells induced by TSH in culture. IGF-1 has also been reported to increase the expression level of TSHR in GO-OFs (63). Conditional knockout of the IGF-1R gene in the thyroid gland significantly reduces the response to TSH (64). Krieger et al. (65) showed that IGF-1R activation by GO immunoglobulins (Igs) occurs *via* TSHR/IGF-1R crosstalk after binding to TSHR and not through direct binding to IGF-1R. Research has revealed that nuclear FOXO transcription factors serve as convergence points for the IGF-1R and TSHR signalling pathways in GO (66, 67). FOXO1 and FOXO3a act as repressors to prevent excessive fat and HA production in OFs, respectively (66). FOXOs may be an alternative target for nonimmunosuppressive therapy.

**Figure 2:**
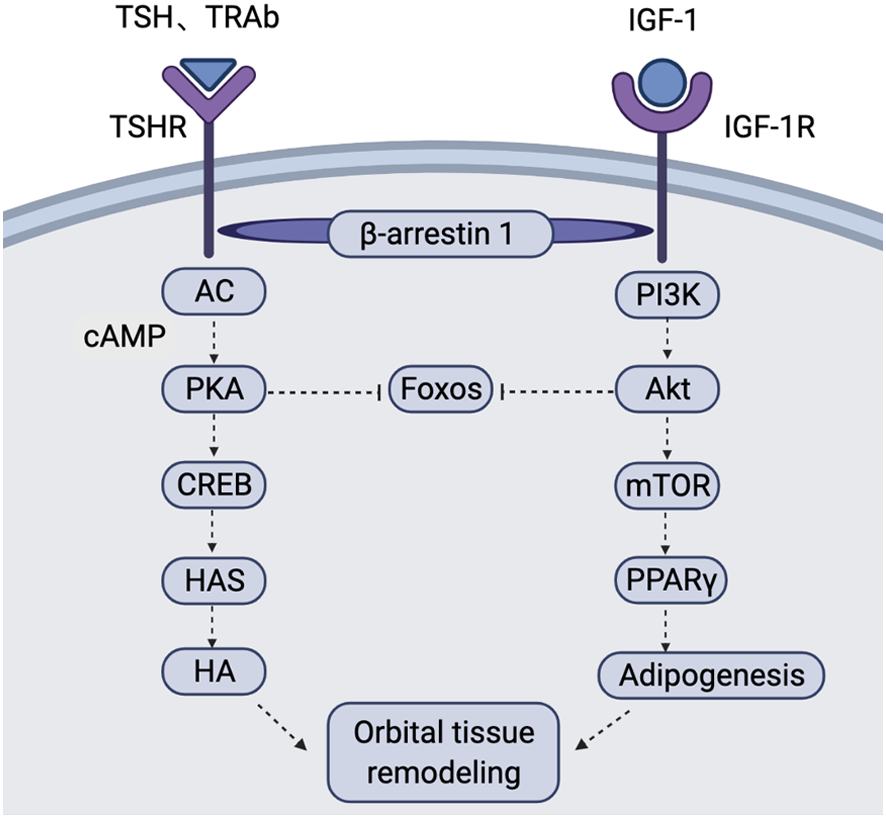
Schematic of the TSHR and IGF-1R signalling pathways in the pathogenesis of GO In OFs and OAs of GO patients, TSH and TRAb bind to TSHR to induce activation of AC. This causes an increase in cAMP production and activates PKA. Activated PKA inhibits FOXOs and activates the transcription factor CREB. CREB further acts on the promoters of HASs to increase HA production. Binding of IGF-1 to IGF-1R activates PI3K, resulting in AKT activation. Activated AKT inhibits FOXOs and activates mTOR to promote adipogenesis *via* PPARγ. TSHR/IGF-1R crosstalk depends on β-arrestin 1 acting as a scaffold, leading to interaction of the two signalling pathways. This figure is adapted from Draman Mohd Shazli,Zhang Lei,Dayan Colin et al. Front Endocrinol (Lausanne), 2021, 12: 739994. AC, adenylate cyclase; AKT, protein kinase B; cAMP, cyclic adenosine monophosphate; CREB, cAMP response element-binding protein; FOXOs, Forkhead box O; GO, Graves’ orbitopathy; HA, hyaluronan; HASs, hyaluronan synthases; mTOR, mammalian target of rapamycin; OAs, orbital adipocytes; OFs, orbital fibroblasts; PI3K, phosphatidylinositol-3-kinase; PKA, protein kinase A; PPARγ, peroxisome proliferator activator gamma; TRAb, thyrotropin receptor antibody; TSH, thyrotropin; TSHR, thyrotropin receptor.

Nevertheless, the manner in which TSHR and IGF-1R interact is not clear. They may interact by activating overlapping signalling pathways or physically through direct heterodimerization. Additionally, β-arrestin 1 and β-arrestin 2 have been reported to be key factors in the regulation of TSHR-mediated signalling (68): β-arrestin 2 plays a major role in TSHR desensitization (69), and β-arrestin 1 is primarily involved in activating TSHR signalling (70). Krieger et al. (71) showed that β-arrestin 1 is a member of a preformed complex that includes TSHR and IGF-1R and that its presence is necessary for stability of the complex in GO-OFs, human thyrocytes and U2OS cells. Knockdown of β-arrestin 1 reduces TSHR/IGF-1R colocalization, blocks TSHR/IGF-1R crosstalk and prevents teprotumumab-mediated inhibition of HA production by GO-Igs and M22 (72). These findings support a model of TSHR/IGF-1R crosstalk that may be a general mechanism underlying GPCR/RTK crosstalk that depends on β-arrestin 1 (**Figure 2**).

## Autoantibodies in GO pathogenesis

### TRAbs

Three types of TRAbs have been identified in recent decades: stimulatory (TSAbs), blocking (TBAbs) and neutral TRAbs (73). Although all three types of TRAbs can be found in patients with GD, TSAbs serve as a marker of the disease (74). Considerable experimental evidence supports the concept that the shedding of the TSHR A subunit in genetically susceptible individuals is a contributing factor to the induction and/or affinity maturation of pathogenic TSAbs, which are the direct cause of GD (75). In recent years, clinical research on TRAbs has gradually moved from general to detailed and now includes research on TSAbs, TBAbs and their titres. A retrospective study confirmed that 70% of euthyroid patients with GO are positive for both TSBAbs and TSAbs at their initial visit (76). A recent study detected TSAb activity in 85 of 91 (93.4%) patients with GO (P < 0.001). The sensitivity rates for differentiating between clinically active versus inactive and mild versus moderate-to-severe GO are both 100% for TSAbs. This finding suggests that TSAbs are highly prevalent in patients with GO and exhibit superior clinical features and predictive potential (77). One study showed a shift from TSAbs to TBAbs in 8 GD patients with/without GO during methimazole treatment that led to remission (78). According to *in vitro* experiments, TSAbs exhibit selectivity in activating TSHRs, as TSAbs from patients with GO are more effective in stimulating OFs, and TSAbs from patients with GD are more effective in stimulating thyrocytes (79). The study by Kahaly et al. (80) confirmed that TRAb titres, as determined by dilution analysis, significantly differentiate between patients with GD and patients with GO. Although these data are potentially interesting and suggestive of a pathogenic role in GO, these experiments have not been compared with the standard bioassay for TSAbs (CHO cells/cAMP stimulation). Therefore, further confirmation is needed. TRAb binding to TSHR induces GAG production through cAMP and the PI3K/AKT signalling pathway, which overlaps with the signalling pathway downstream of IGF-1R. A recent study found that TSAbs are able to induce IGF-1R phosphorylation and initiate both TSHR and IGF-1R signalling in human and mouse fibroblasts. These findings indicate that TSAbs enhance IGF-1R activity and contribute to retroorbital cellular proliferation and inflammation (58). Kumar et al. (81) showed that treatment of orbital preadipocytes from patients with GO with M22, a human monoclonal TSAb, during adipocyte differentiation results in enhanced mRNA expression levels of IL-6. Furthermore, treatment of orbital cultures with M22 after adipocyte differentiation increases secretion of the IL-6 protein into the medium. These results suggest that M22 increases IL-6 expression in orbital preadipocyte fibroblasts and increases IL-6 release by mature adipocytes and that circulating TRAbs might play a direct role in the clinical activity of GO.

### Anti-IGF-1R antibodies

A role for IGF-1R in the pathogenesis of GO has been proposed, but the presence and function of IGF-1R antibodies (IGF-1R Abs) are controversial (82). The findings of Marcus-Samuels et al. (83) demonstrated that knockdown of IGF-1R causes a 6.3-65% decrease in IGF-1-stimulated pAKT but has no effect on GO-Ig stimulation of pAKT, suggesting that GO-Igs contain factors that stimulate pAKT formation but that this factor does not directly activate IGF-1R. It was concluded that there is no evidence of stimulating IGF-R Abs in patients with GO. However, other studies have shown the presence of IGF-1R Abs. A study from Weightman DR et al. first demonstrated that IgG prepared from patients with GD with or without overt ophthalmopathy interacts with IGF-1 binding sites on OFs, suggesting that antibodies may occur in GD that bind to IGF-1R (84). Minich et al. (85) detected IGF-IR Abs in 10 serum samples from control subjects (11%) and 60 serum samples from patients with GO (10%). They found a similar prevalence of IGF-1R Abs in GO patients and controls, with an obvious lack of correlation between IGF-1R Ab concentrations and CAS or NOSPECS status. Therefore, this study suggested that IGF-1R Abs in the blood of patients with GO do not exacerbate the disease and are in fact antagonistic. In addition, this study confirmed that IGF-IR Abs fail to stimulate autophosphorylation of IGF-1R and instead inhibit IGF1-induced signalling in HepG2 hepatocarcinoma cells (85). Another study showed that regardless of the presence of GO, a quarter of patients with GD have IGF-IR Abs in their sera. In patients with GO, there is no relationship between GO severity and IGF-1R Ab levels, although the levels of these antibodies correlate inversely with CAS (86). Lanzolla et al. (87) further designed a cross-sectional investigation to measure IGF-1R Ab levels in patients with GD, with or without GO, who underwent radioiodine therapy followed by glucocorticoid treatment and found higher IGF-1R Ab levels in GD patients without GO than in those with GO. These results suggest a putative protective role for IGF-1R Abs with respect to the development of GO, which is consistent with the beneficial effects of teprotumumab on GO (87). In contrast, Varewijck et al. (88) found that GO patients with high TSAb titres are more likely to have stimulatory IGF-1R Abs. In summary, whether IGF-1R Abs exist and their role deserve further study.

## Treatments that target TSHR and IGF-1R

### TSHR antagonists

Because fibroblast production of cAMP, pAKT, and HA is activated *via* TSHR signalling, future inhibitory therapies targeting TSHR may be effective for GO. K1-70, a human monoclonal autoantibody, has considerable promise as a new drug for blocking the actions of thyroid stimulators on TSHR. In a recent case report, K1−70 was injected intramuscularly at 3 weekly intervals in a patient with follicular thyroid cancer, GD and GO, TSAb activity in the serum decreased, and proptosis and inflammation improved (89). In a phase I clinical trial, 18 patients with stable GD that were given antithyroid drug medication received a single intramuscular dose of 25 mg or a single intravenous dose of 50 mg or 150 mg of K1-70. In these patients, the thyroid function, GD and GO clinical manifestations improved after the injection. No immunogenic reaction, death or serious adverse events occurred (90). Currently, several small TSHR antagonists have also been studied *in vitro* in human samples. For example, in an *in vitro* human experiment, a small TSHR antagonist (NCGC00229600, also called ANTAG2) inhibited basal cAMP, pAKT, and HA production and that stimulated by TSAb (M22 and MS-1) and bTSH in primary cultures of undifferentiated GO-OFs (91). Another *in vitro* study found that a low-molecular-weight TSHR antagonist (Org-274179-0) completely blocks cAMP production in differentiated GO-OFs induced by human recombinant GD-IgG, TSH or M22 (92). Marcinkowski et al. (93) showed that a novel, highly selective inhibitor of TSHR (S37a) inhibits TSHR activation not only *via* TSH but also *via* stimulatory monoclonal TRAbs M22 (human), KSAb1 (murine) and the allosteric small-molecule agonist C2 in HEK293 cells expressing TSHR *in vitro*. Moreover, the addition of 50 µM S37a inhibited cAMP accumulation induced by sera from patients with GO by 50‐60% compared to bTSH-treated stable HEK-TSHR cells. *In vivo* experiments in mice are also being studied and may provide new ideas for treating GO. The findings of *in vivo* experimental studies by Fassbender et al. (94) demonstrate that novel peptides distinctly reduce serum thyroxine levels, thyroid size, retro-orbital fibrosis and tachycardia in Ad-TSHR289-immunized mice. In immunologically naïve mice, the administration of peptides did not induce any immune response. This evidence also indicates that intravenous administration of TSHR-derived cyclic peptide 19 to Ad-TSHR-immunized mice significantly improves thyroid function, the levels of TRAbs and acidic mucins and the collagen content in orbital tissue, offering a potential novel therapeutic approach for GD and GO (95). A recent study indicated that GO mice intraperitoneally injected with siRNA targeting TSHR exhibited marked improvements in weight loss, serum thyroxine (T4) levels, TSAb levels, TSBAb levels and thyroid uptake of 99mTcO4 (96). Potential novel therapies for GO that directly target TSHR are provided in **Table 1**.

**Table 1:** Potential novel therapies for GO that directly target TSHR.

Type	Inhibitor	Model		Main effect		Reference
Human monoclonal autoantibody	K1–70	Human (a patient with FTC, GD and GO) *in vivo*		Decreased the TSAb activity in the serum and improved proptosis and inflammation		(89)
	K1–70	Human (in a phase I clinical trial, n=18) *in vivo*		Improved thyroid function, GD and GO clinical manifestations		(90)
Small TSHR antagonists	NCGC00229600 (ANTAG2)	Human (GO-OFs) *in vitro*		Inhibited basal cAMP, pAKT, and HA production and that stimulated by TSAb and bTSH		(91)
	Org-274179-0	Human (GO-OFs) *in vitro*		Blocked the cAMP production of differentiated GO-OFs induced by human recombinant TSH, GD-IgG, or M22		(92)
	S37a	HEK cells (stably expressing TSHR) *in vitro*		Inhibited the cAMP accumulation induced by sera from patients with GO		(93)
Peptide	TSHR-derived peptide 836 13-mer, peptide 19, peptide 12	Mice (Ad-TSHR289-immunized mice) *in vivo*		Reduced thyroid size, serum thyroxine levels, retro-orbital fibrosis, and tachycardia		(94)
	TSHR-derived cyclic peptide 19 (P19)	Mice (Ad-TSHR-immunized mice) *in vivo*		Improved thyroid function, TRAbs and orbital mucine/collagen content		(95)
siRNA	siRNA targeting TSHR	Mice (BALB/c mouse model of GD) *in vivo*		Improved weight loss, T4 levels, TSAb levels, TSBAb levels and thyroid uptake of 99mTcO4		(96)

cAMP, cyclic adenosine monophosphate; GO, Graves’ orbitopathy; GO-OFs, orbital fibroblasts from patients with Graves’ ophthalmopathy; HA, hyaluronan; TSAb, TSHR stimulatory antibody; TSH, thyrotropin; TSHR, thyrotropin receptor.

### IGF-1R blockers

Recently, the FDA-approved drug teprotumumab (a human monoclonal anti-IGF-1R Ab) was identified as a breakthrough drug for treating active GO. Teprotumumab reduces proptosis and the need for orbital decompression surgery. Teprotumumab mainly inhibits the IGF-1R pathway through two mechanisms. First, upon binding to the cysteine-rich domain of human IGF-1R, teprotumumab blocks the binding pocket for both endogenous ligands (IGF-1 and IGF-2) and prevents them from activating the signalling cascade. Second, binding of teprotumumab induces internalization and subsequent degradation of IGF-1R, leading to a 95% reduction in cell surface-accessible IGF-1R (97, 98). Teprotumumab does not bind to TSHRs and inhibits IGF1R-dependent M22-induced HA production, which is mediated by TSHR/IGF-1R crosstalk but not IGF-1R-independent M22 stimulation (72). Fibrocytes from patients with GD display a robust reduction in surface IGF-1R and TSHR expression after teprotumumab treatment (99).

A randomized, placebo-controlled, multicentre, double-masked trial was performed to investigate the efficacy of teprotumumab in patients with active, moderate-to severe GO (100). Eighty-eight patients were randomly assigned to receive the active drug or placebo administered intravenously once every 3 weeks for a total of eight infusions. Compared with patients who received the placebo, those who received teprotumumab showed a response at week 24. The therapeutic effects were rapid; at week 6, 18 of the 43 patients in the teprotumumab group and 2 of the 45 patients in the placebo group showed a response. These data indicate that teprotumumab therapy provides clinical benefits to patients with active, moderate-to-severe GO by reducing proptosis and CAS and by improving quality of life (100).

Another randomized, double-masked, placebo-controlled, phase 3 multicentre trial was carried out in 2020. Forty-one patients were assigned to the teprotumumab group and received intravenous infusions of teprotumumab (10 mg/kg for the first infusion and 20 mg/kg for subsequent infusions). Forty-two patients were assigned to the placebo group and received intravenous infusions of placebo once every 3 weeks for 21 weeks. The last trial visit for this analysis occurred at week 24, at which the percentage of patients showing a proptosis response was higher in the teprotumumab group. All secondary outcomes, including overall response, the proportion of patients with CAS of 0 or 1, the mean change in proptosis, the diplopia response, and the mean change in Graves’ ophthalmopathy-specific quality-of-life overall score, were significantly better after treatment with teprotumumab than with placebo. These findings demonstrate that among patients with active GO, teprotumumab resulted in better outcomes than placebo with respect to proptosis, CAS, diplopia, and quality of life. The most common adverse events (AEs) reported with teprotumumab included muscle spasms, alopecia, nausea, diarrhoea, hearing impairment, fatigue and hyperglycaemia, but they were mild-moderate during treatment. Serious AEs, such as Hashimoto’s encephalopathy, were uncommon (101). A summary of two teprotumumab clinical trials for GO is shown in **Table 2**.

**Table 2:** Summary of two teprotumumab clinical trials for GO.

Reference	(100)	(101)
Group	Teprotumumab (n=43) Placebo (n=45)	Teprotumumab (n=41) Placebo (n=42)
Primary end points	Reduction of 2 points or more in the CAS at week 24 Reduction of 2 mm or more in proptosis at week 24	Proptosis response at week 24
Secondary end points	At week 6, 12, 18, and 24 Proptosis CAS CAS of 0 or 1 GO-QOL Diplopia	Overall response at week 24 CAS of 0 or 1 at week 24 The mean change in proptosis across trial visits Diplopia response at week 24 The mean change in overall score on GO-QOL across trial visits
Result for primary end points	In total, 29 of 42 patients who received teprotumumab (69%), compared with 9 of 45 patients who received placebo (20%), had a response at week 24	At week 24, the percentage of patients with a proptosis response was higher with teprotumumab than with placebo
Result for secondary end points	At week 6, a total of 18 of 42 patients in the teprotumumab group (43%) and 2 of 45 patients in the placebo group (4%) had a response	All secondary outcomes were significantly better with teprotumumab than with placebo
Conclusion	In patients with active ophthalmopathy, teprotumumab was more effective than the placebo in reducing proptosis and the CAS and improving the patients’ quality of life	Among patients with active thyroid eye disease, teprotumumab resulted in better outcomes with respect to proptosis, CAS, diplopia, and quality of life than placebo

GO, Graves’ orbitopathy; CAS, Clinical Activity Score; GO-QOL, Graves’ ophthalmopathy–specific quality-of-life questionnaire.

Currently, high-dose intravenous methylprednisolone combined with oral mycophenolate is the preferred therapy for moderate-to-severe and active GO, as cited by the 2021 EUGOGO. Other biological agents, such as tocilizumab (a humanized monoclonal antibody against IL-6R) (102–109), rituximab (a monoclonal antibody that targets CD20+ B cells) (108, 110–116), and adalimumab (a TNF-α blocking monoclonal antibody) (117), have proven to be useful and safe therapeutic options as treatments in refractory GO. These agents have produced promising results, including improved proptosis and diplopia and reduced CAS scores. Other biological agents of GO are listed in **Table 3**.

**Table 3:** Other clinical therapeutic targets for GO.

Agent	Type	Target	Main effect	Research method	Clinical conclusion	Adverse reactions/Relapse	References
Tocilizumab	Humanized monoclonal antibody	IL-6 receptor	Inhibition of the pro-inflammatory effects of IL-6	Retrospective case reports (n=3)	Improved ocular symptoms (diplopia and proptosis) and functional prognosis in severe or corticosteroid-resistant GO.	One relapse approximately two months after the end of the treatment.	(102)
				Double-masked RCT (n=32)	Improved activity and severity in corticosteroid-resistant GO.	Moderate increase in transaminases (n=1); acute pyelonephritis (n=1).	(103)
				Observational single-centre study (n=10)	Rapidly effective and well-tolerated in patients with GC-refractory GO.	Neutropenia, hyperlipidaemia, and infections (n=4); nearly one-third developed cancer during the follow-up.	(104)
				Retrospective longitudinal study (n=54)	Provided a significant benefit to patients with active moderate-to-severe steroid-resistant GO.	–	(105)
				Open-label multicentre study (n=48)	A useful and safe therapeutic option in refractory GO treatment.	No serious adverse events.	(106)
				Retrospective longitudinal study (n=9)	As a therapy for the inflammatory phase of GO.	Elevated cholesterol (n=1). No patients had recurrence of active disease.	(107)
				Retrospective longitudinal study (n=7)	A significant improvement in the CAS, visual acuity, diplopia, and proptosis.	Significant relapse (n=1).	(108)
				A case series report (n=8)	A therapeutic option for glucocorticoid-resistant orbitopathy.	–	(109)
Rituximab	Chimeric human and mouse monoclonal antibody	CD20	Immunosuppression through B-cell depletion	Retrospective longitudinal study (n=9)	A significant improvement in the CAS, visual acuity, diplopia, and proptosis.	Significant relapse (n=4).	(108)
				Retrospective analysis (n=15)	Sustained anti-inflammatory effect in most patients with active GO resistant to conventional treatment.	No significant effect.	(110)
				Retrospective audit (n=12)	An efficacious, well-tolerated and safe treatment for active GO; reduced disease activity and allowing reduced administration of systemic steroids.	A transient infusion-related rash (n=4).	(111)
				Retrospective case (n=14)	A well-tolerated treatment with a good safety profile but offered limited and partial improvement for active moderate-to-severe GO with a long duration of disease.	Moderate adverse event (n=1).	(112)
				A post hoc analysis of two open label studies and one prospective trial randomized (n=40)	500 mg RTX in the treatment of active moderate-to-severe GO.	–	(113)
				Open-label prospective study (n=17)	A dose of 100 mg RTX is effective in patients with active moderate-to-severe GO.	Cytokine release syndrome (n=1).	(114)
				Multicentre retrospective study (n=40)	Effective as a second-line treatment for the inflammatory component of GO, especially if the disease is highly active and recent.	Cytokine release syndrome (n=1).	(115)
				Double-blind RCT (n=32)	A better therapeutic outcome in active moderate-to-severe GO, eye motility outcome, visual functioning of the quality of life assessment, and the reduced number of surgical procedures.	–	(116)
Adalimumab	TNF-α blocking monoclonal antibody	TNF-α	Inhibition of HA production and inflammation	Retrospective study (n=10)	Has a role in the treatment of active GO with prominent inflammatory symptoms.	Sepsis (n=1).	(117)

GO, Graves’ orbitopathy; HA, hyaluronan; RCT, randomized clinical trial; GC, glucocorticoids; CAS, Clinical Activity Score; RTX, rituximab.

## Conclusions

The following important points were obtained from the review. In addition to CD34+ OFs, CD34- OFs are crucial for GO pathogenesis and may be involved in HA formation. CD34- OFs expressing Slit2 suppress the phenotype of CD34+ OFs, and β-arrestin 1 is involved in TSHR/IGF-1R crosstalk. In recent years, research on TRAbs has gradually shifted to TSAbs and TBAbs, but the existence and role of IGF-1R Abs remain unclear, and further exploration is warranted. The results of clinical trials targeting TSHR inhibition are expected. Teprotumumab has become the latest second-line treatment for GO. Tocilizumab and rituximab hold great promise in the future management of GO and can be useful in cases if intolerance or resistance to standard immunosuppressive treatment.

## Author contributions

This review was written by XC. All authors contributed to the article and approved the submitted version.
